# Genetic diversity of Newcastle disease viruses circulating in wild and synanthropic birds in Ukraine between 2006 and 2015

**DOI:** 10.3389/fvets.2023.1026296

**Published:** 2023-01-19

**Authors:** Iryna V. Goraichuk, Anton Gerilovych, Vitaliy Bolotin, Olexii Solodiankin, Kiril M. Dimitrov, Oleksandr Rula, Nataliia Muzyka, Oleksandr Mezinov, Borys Stegniy, Olena Kolesnyk, Mary J. Pantin-Jackwood, Patti J. Miller, Claudio L. Afonso, Denys Muzyka

**Affiliations:** ^1^National Scientific Centre, Institute of Experimental and Clinical Veterinary Medicine, Kharkiv, Ukraine; ^2^Exotic and Emerging Avian Viral Diseases Research Unit, Southeast Poultry Research Laboratory, US National Poultry Research Center, Agricultural Research Service, USDA, Athens, GA, United States; ^3^The F.E. Falz-Fein Biosphere Reserve “Askania Nova”, National Academy of Agrarian Sciences of Ukraine, Askania-Nova, Kherson Oblast, Ukraine; ^4^Department of Zoology, H.S. Skovoroda Kharkiv National Pedagogical University, Kharkiv, Ukraine

**Keywords:** NDV, *Avian orthoavulavirus 1*, surveillance, sequencing, bird migration, synanthropic, pigeon, Ukraine

## Abstract

Newcastle disease virus (NDV) infects a wide range of bird species worldwide and is of importance to the poultry industry. Although certain virus genotypes are clearly associated with wild bird species, the role of those species in the movement of viruses and the migratory routes they follow is still unclear. In this study, we performed a phylogenetic analysis of nineteen NDV sequences that were identified among 21,924 samples collected from wild and synanthropic birds from different regions of Ukraine from 2006 to 2015 and compared them with isolates from other continents. In synanthropic birds, NDV strains of genotype II, VI, VII, and XXI of class II were detected. The fusion gene sequences of these strains were similar to strains detected in birds from different geographical regions of Europe and Asia. However, it is noteworthy to mention the isolation of vaccine viruses from synanthropic birds, suggesting the possibility of their role in viral transmission from vaccinated poultry to wild birds, which may lead to the further spreading of vaccine viruses into other regions during wild bird migration. Moreover, here we present the first publicly available complete NDV F gene from a crow (genus *Corvus*). Additionally, our phylogenetic results indicated a possible connection of Ukrainian NDV isolates with genotype XXI strains circulating in Kazakhstan. Among strains from wild birds, NDVs of genotype 1 of class I and genotype I of class II were detected. The phylogenetic analysis highlighted the possible exchange of these NDV strains between wild waterfowl from the Azov-Black Sea region of Ukraine and waterfowl from different continents, including Europe, Asia, and Africa.

## 1. Introduction

Newcastle disease virus (NDV), or *Avian orthoavulavirus 1*, is a member of the recently separated subfamily *Avulavirinae* in the *Paramyxoviridae* family (1). NDV is capable of infecting a wide range of bird species and is of great economic importance to both large poultry enterprises and private farms. Since the discovery of the first NDV in the 1920s, an array of data has accumulated illustrating the genetic and pathogenic diversity of the viral strains (2). According to the Terrestrial Manual (World Organization for Animal Health, formerly the Office International des Epizooties), (WOAH, founded as OIE) Terrestrial Manual, NDV strains are divided into three main pathogenicity groups: velo-, meso-, and lentogenic (3, 4). Velogenic strains cause hemorrhages, reduced egg production, intestinal lesions, and neurological symptoms with high mortality in infected chickens. Mesogenic strains cause disease with respiratory or neurological symptoms but little mortality. Lentogenic strains can cause subclinical respiratory or intestinal infections and are considered low-virulent. *Avian orthoavulavirus 1* strains are divided into two classes based on the nucleotide sequence of the fusion (F) protein gene. Class I consists mainly of lentogenic strains isolated from wild birds worldwide and includes only one genotype (5). Class II contains 21 genotypes with velo-, meso- and lentogenic strains that have been detected in a wide variety of host species around the world (6).

The fusion protein is an important determinant of NDV pathogenicity (7, 8). It is synthesized as an inactive precursor (F0), which is proteolytically cleaved by host proteases into two polypeptides (F1 and F2) for the virus particles to be infectious. The efficiency of proteolytic cleavage is dependent on the host cell and the virus strain (9–11). F proteins of velogenic and mesogenic NDV virus strains are characterized by the presence of multiple basic amino acids at the F0 cleavage sequence which are recognized by ubiquitous host cell proteases (12–14). In contrast, the F0 protein of lentogenic strains has a monobasic cleavage site, which is cleavable by a restricted number of certain host proteases. These differences in the F gene sequences which correlate with different virulence phenotypes are prime targets for the development of molecular biological approaches to identify and characterize NDV isolates. However, the viral population may acquire mutations and adaptive changes in response to different pressures by the host's immune system (15, 16). Together with large genetic diversity it can affect real-time PCR specificity for NDV detection, particularly virulence determination (17, 18) and vaccine efficacy (19, 20) since both are typically based on the genetic sequence of the F protein, most often affected by vaccine pressure.

Although a wealth of information about this virus has accumulated to date, the role of wild birds in the global circulation and epidemiology of certain NDV genotypes remains unclear. The expansion of the surveillance program for *Avian orthoavulavirus 1* will improve our understanding of the global epidemiological picture, as well as help effectively prevent Newcastle Disease (ND). A large-scale study of the potential host range of NDV among wild birds in Africa showed the year-round presence of viruses, as well as their phylogenetic relationship with strains of domestic birds in the study area (21). Currently, in the territory of Eurasia, few studies of the potential host range of *Avian orthoavulavirus 1* have been carried out, and the seasonality of infection has not been established. Even though there are many reports of NDV isolated from wild birds in this region, more than 30 countries remain unstudied or with limited data (22–29). Therefore, studies performed at the major stopping point locations for migratory birds can be of great contribution toward gaining more knowledge on the potential host range of NDV therein.

Ukraine occupies a unique geographical location in central and Eastern Europe, where the West Asia-East Africa flyways of wild migratory birds cross the Black Sea-Mediterranean, and East Atlantic flyways (30, 31). The natural conditions such as climate and the abundance of wetlands with an area of more than 590,000 ha in Ukraine contribute to the year-round presence of a large number of wild birds. This is especially evident during the period of seasonal migrations and wintering, when numerous wild bird species pass from North Asia and Europe to the Mediterranean, Africa, and Southwest Asia, and also cross from the Baltic and Caspian Seas to the Black and Mediterranean Seas, and from western Siberia and Kazakhstan to Western Europe and North Africa (32). The Azov-Black Sea region is one of the densest territories of Eastern Europe from an ornithological point of view. This region is historically an area of nesting, flight, migratory stops, and wintering for many bird species. Therefore, a large number of waterfowl and waterbirds from the Central part of Eurasia winter in the Azov-Black Sea region of Ukraine or stop there during migrations. As a result, wild bird populations from Ukraine and West Europe can interact with birds from Asia and Africa (33).

Domestic poultry also plays a role in the circulation of *Avian orthoavulavirus 1*. According to data from the United Nations Food and Agriculture Organization (FAO), the total poultry presence in the world (chickens, ducks, turkeys, geese, and guinea fowl) was ~35 billion birds in 2020 (34). Chickens accounted for 94% of the world's poultry population with about 46% of these located in the territory of Asia. To help keep ND under control in developed countries, industrial poultry farms are recommended to vaccinate their birds (mainly chickens and turkeys) and isolate them from the external environment to avoid contact with wild and synanthropic bird species (35, 36). But cases of possible transmission of the NDV vaccine strain from poultry to wild birds have been described (23, 37, 38). There are also reports of velogenic strains in wild birds (39). Currently, it is not well studied if lentogenic strains of NDV from wild birds may cause respiratory infection in poorly vaccinated poultry birds following exposure. However, a few cases of the change in the virus virulence were well-documented. Retrospective studies showed that cumulative mutations at the fusion protein cleavage site acquired through natural transmission at the poultry farms lead to change in the virulence of originally lentogenic virus during NDV outbreak of genotype I in Australia (40–42). Another study demonstrated an initially non-pathogenic strain from a wild duck acquiring pathogenicity through passaging in chickens (43).

Currently, the issue of the introduction and circulation of lentogenic NDV vaccine strains into the wild bird population is under consideration and discussion, thus it is important to assess the presence of vaccine spillovers to wild and synanthropic birds as well (23). Therefore, this article presents surveillance data of *Avian orthoavulavirus 1* circulation in a natural reservoir from eight different regions in the South and East of Ukraine, including migratory bird's stopping points located in the Azov-Black Sea region. Part of these results was previously reported (5, 23, 27, 29, 44).

## 2. Materials and methods

### 2.1. Sample collection

**Figure 1:**
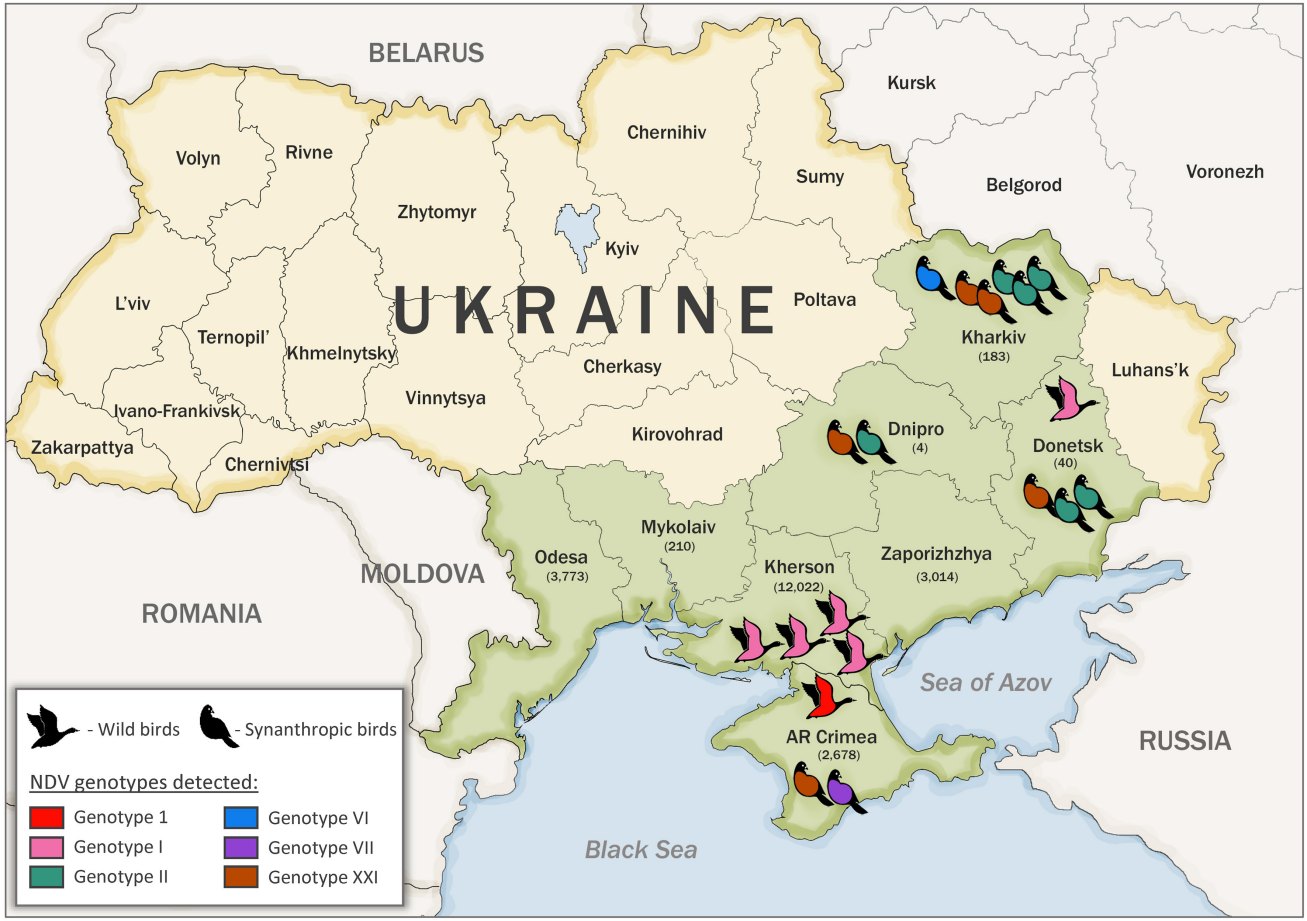
Sample collection number, bird type, and location in Ukraine. Sampled regions are indicated in green color. The number of collected samples are indicated in parenthesis under the name of each region. Genotypes detected in this study are shown in red (genotype 1, class I), pink (genotype I, class II), teal (genotype II, class II), blue (genotype VI, class II), purple (genotype VII, class II), and brown (genotype XXI, class II).

Samples from wild and synanthropic birds were collected in Ukraine between 2006 and 2015 during active and passive surveillance (29, 45). Wild birds were defined as those occurring in a natural habitat other than poultry and synanthropic birds were defined as undomesticated birds that live in close association with people. A total of 21,924 samples were collected, among them, 21,854 samples were collected from 103 species of wild birds during active surveillance and 70 samples from synanthropic birds during passive surveillance. The sampling sites were located in the Azov-Black Sea region [Kherson, Mykolaiv, Odesa, Zaporizhzhya, and the Autonomous Republic (AR) of Crimea regions] and the East of Ukraine (Donetsk, Dnipro, and Kharkiv regions) (Figure 1). The samples' background data, including bird species and location, were recorded (Supplementary Tables S1, S2).

Sampling from wild birds was carried out in cooperation with ornithologists, who helped identify the bird species. Cloacal swabs were collected from apparently healthy live-trapped and hunted wild birds. Fresh feces were collected from places of mass bird accumulation. Feces were collected only if the origin and type of bird had been established. Immediately after sampling, samples of the biological material from wild birds were placed in tubes with a transport medium (Hank's balanced salt solution containing 0.5% lactalbumin, 10% glycerol, 200 U penicillin, 0.200 mg streptomycin, 100 U polymyxin, 0.250 mg gentamicin, and 50 U nystatin per ml) then stored and transported on ice to the laboratory and further stored in liquid nitrogen.

Samples of spleen, brain, liver, and intestines were collected from dead synanthropic birds (pigeons and gray crows) in Ukraine between 2006 and 2015. All dead synanthropic birds were found in locations of their natural habitat within cities in Kharkiv, Dnipro, Donetsk, Odesa, and AR Crimea regions. Samples were chilled at 4°C, transported to the lab, and stored at −80°C. Before analysis, samples were thawed and suspended in transport media (10% w/v).

### 2.2. Virus isolation and identification

Each fecal/cloacal swab medium or tissue suspension supernatant was inoculated (0.2 ml) into five 9- to 11-day-old specific-pathogen-free (SPF) embryonated chicken eggs (ECEs) using standard methods as described previously (46, 47). Allantoic fluids from all inoculated ECEs were harvested and tested for hemagglutination activity with chicken red blood cells using a hemagglutination assay (HA) (48). All HA-positive samples were analyzed in the hemagglutination inhibition (HI) assay using reference antisera to influenza A virus subtypes H1–H16 and avian paramyxoviruses (APMV-1–APMV-9) according to previous recommendations (47, 49, 50). The identification of avian influenza viruses and APMV-4, APMV-6, APMV-7, and APMV-13 have been previously described (29, 32, 51, 52).

Pathogenicity evaluation was performed on eleven selected NDV isolates (KF851268–KF851270, KJ914671, KJ914672, KU133362–KU133365, KY042127, KY042128, MZ101338) using the intracerebral pathogenicity index (ICPI) assay on 1-day-old SPF chickens following established procedures at the Southeast Poultry Research Laboratory (SEPRL), U.S. Department of Agriculture (USDA), Athens, GA, USA (3).

### 2.3. RNA extraction and sequencing

All HI-identified NDV isolates were subject to sequencing to determine their genotype and virulence. Viral RNA was extracted from infected allantoic fluids using TRIzol LS (Invitrogen, USA) following the manufacturer's instructions. The nucleotide sequences of the complete coding region of the F protein gene were determined by utilizing the RT-PCR/sequencing approach (53). The amplification reaction was performed using the SuperScript III One-Step RT-PCR System with Platinum Taq DNA Polymerase (Life Technologies, USA) with overlapped primer pairs for sequencing the complete F gene of different NDV genotypes from class I and II as described previously by Miller et al. (24). All RT-PCR products were subjected to electrophoresis in a 1% agarose gel (0.5X TBE). The appropriately sized DNA bands were excised from the gel and purified using the QuickClean II Gel Extraction Kit (GenScript, USA) and subjected to DNA sequencing. Nucleotide sequencing was performed for 19 samples on an ABI Sanger sequencer (Applied Biosystems, USA) with fluorescent dideoxy-nucleotide terminators at SEPRL, USDA, Athens, GA, USA. Sequence editing and assembly were performed using the SeqMan software of the LaserGene package (DNASTAR, USA).

### 2.4. Phylogenetic analysis

Genotype and sub-genotype identification were based on the phylogenetic topology and evolutionary distances between different taxonomic groups using a pilot dataset as described by Dimitrov et al. (5). Multiple sequence alignments of the NDV complete F gene sequences were produced using the MAFFT version 7 software (54). The pilot tree of class I and II NDV isolates (*n* = 98) was constructed using the Maximum-likelihood method based on the General Time-Reversible (GTR) model with a discrete gamma distribution (+G) and allowing for invariant sites (+I) with statistical analysis based on 1,000 bootstrap replicates, as implemented in MEGA7 (55). The tree was drawn to scale, with branch lengths measured in the number of substitutions per site. For all analyses, the codon positions included were 1^st^, 2^nd^, 3^rd^, and non-coding, and all positions containing gaps and missing data were eliminated. A total of 1,661 positions were included in the pilot analysis of the complete F gene dataset.

For each genotype detected (1 of class I and I, II, VI, VII, and XXI of class II), more detailed phylogenetic trees were constructed using the most closely related sequences detected in BLAST. The Roman numerals presented in the name of each sequence in the phylogenetic tree represent the respective sub-genotype, followed by the GenBank accession number, host name, country of isolation, strain designation, and year of isolation (if available).

The complete F gene data set used for the phylogenetic analysis was also used to estimate the average evolutionary distances comparing Ukrainian NDV isolates to other relative strains. Pair-wise analysis was conducted using the maximum composite likelihood model using MEGA7 software (56). The rate variation among sites was modeled with a gamma distribution (shape parameter = 4).

## 3. Results

### 3.1. Geographic distribution of the viruses sequenced

The serological examination of biological material collected from 21,854 wild and 70 synanthropic birds belonging to 105 species and 11 different orders was conducted during 2006–2015. The largest number of samples was collected from birds of the order *Anseriformes* (15,013 samples), followed by *Charadriiformes* (4,737 samples), and *Passeriformes* (1,562 samples). The main sampling sites (~99% of the samples) for wild birds were located in the Azov Black Sea region. This region is the meeting point of the transcontinental migration routes of different wild birds from Siberia, Africa, Europe, and Asia. The rest of the biological samples were collected in the eastern region of Ukraine (Figure 1). NDV was identified in 31 (0.14%) samples from asymptomatic wild birds and 24 (34.29%) dead synanthropic birds by the HI test, part of which was previously reported (22, 29). All three overlapping regions of the complete F gene were successfully amplified by RT-PCR for 18 isolates, previously classified as NDV based on the serological HI test (Table 1). Two complete F genes of different NDV genotypes were amplified and sequenced for one isolate, which brought the total number of obtained sequences to 19. Some of the viruses were sequenced and previously published (5, 23, 27, 29, 44). Sequenced NDV isolates were obtained from dead synanthropic birds and asymptomatic wild waterfowl in different regions of Ukraine (Table 1). Twelve NDV isolates were attained from synanthropic birds (pigeons and crows). Isolates from synanthropic birds were obtained from four different regions: three originated from the Kharkiv region and one each from the Dnipro region, the Donetsk region, and the Autonomous Republic of Crimea (Figure 1). The six isolates from wild birds all came from the Kherson region in the south of Ukraine.

**Table 1:** Background information data for NDV isolates recovered in Ukraine between 2006 and 2015.

**Species**	**Class/sub-genotype**	**Region**	**Isolate**	**Year of isolation**	**ICPI^a^**	**Cleavage site motif**	**GenBank accession number**
Pigeon	II/II	Kharkiv	Kharkiv/1	2007	1.09	GRQGR↓L	KU133364
Pigeon	II/II	Kharkiv	Kharkiv/2	2007	0.29	GRQGR↓L	KU133365
Pigeon	II/II	Dnipro	Dnipropetrovsk/07	2007	1	GRQGR↓L	KU133363
Pigeon	II/XXI.1.1	Donetsk	Doneck/3/968	2007	1.48	**KRQKR↓F**	KY042128
	II/II					GRQGR↓L	KU133362
Crow	II/II	Kharkiv	Izum/8-15^c^	2007	N/A	GRQGR↓L	MZ101343
Pigeon	II/II	Donetsk	Doneck/10/26-6^c^	2008	N/A	GRQGR↓L	MZ101344
Teal	II/I.2	Donetsk	Krasnooskilsky/5-11	2009	0.08	GKQGR↓L	KF851269
Mallard	I/1.2	AR Crimea	Krasnoperekopsk/18-23-10	2010	0.16	ERQER↓L	KF851268
Ruddy shelduck	II/I.2	Kherson	Askania-Nova/3-20-11^c^	2010	0.05	GKQGR↓L	MZ101338
Pigeon	II/VII.1.1	AR Crimea	Simferopol/2-26-11	2011	N/A	**RRQKR↓F**	KU710277
Ruddy shelduck	II/I.2	Kherson	Askania-Nova/37-15-02	2011	0.76	GKQGR↓L	KF851270
Pigeon	II/XXI.1.1	Dnipro	Dnipropetrovsk/1-18-11^c^	2011	1.15	**KRQKR↓F**	KJ914671
Pigeon	II/XXI.1.1	AR Crimea	Ukromne/3-26-11^c^	2011	1.36	**KRQKR↓F**	KJ914672
Pigeon	II/XXI.1.1	Kharkiv	Kharkiv/23-01/967	2013	1.7	**KRQKR↓F**	KY042127
White-fronted Goose	II/I.2	Kherson	Askania-Nova/72-28-03^c^	2013	N/A^b^	GKQGR↓L	MZ101339
Mediterranean Gull	II/I.2	Kherson	Smalanyi/5-11-07^c^	2013	N/A	GKQGR↓L	MZ101340
Pigeon	II/XXI.1.1	Kharkiv	Kuksov/13-05^c^	2014	N/A	**KRQKR↓F**	MZ101341
Pigeon	II/VI.2.1.1.2.2	Kharkiv	Petruk/15-01^c^	2015	N/A	**RRQKR↓F**	MZ101342

^a^ICPI, intracerebral pathogenicity index.

^b^N/A, not available.

^c^Sequenced in this study.

The virulent cleavage sites are highlighted in bold.

### 3.2. Pathotype characterization

NDV pathogenicity markers were examined for all 19 NDV sequences. The deduced amino acid sequences of the fusion protein cleavage site revealed that sequences of seven Ukrainian isolates from this study presented four basic amino acids at the C-terminus of the F2 protein from residues 112–116 and phenylalanine at residue 117 (Table 1, highlighted in bold), and based on the WOAH definition of vNDV molecular pathotyping (3) they are considered as virulent strains. The cleavage site motifs of these Ukrainian isolates were ^112^KRQKR↓^117^ (*n* = 5) and ^112^RRQKR↓F^117^ (*n* = 2). All seven viruses were isolated from synanthropic pigeons in the Dnipro, Donetsk, Kharkiv, and AR Crimea regions of Ukraine. The rest of the cleavage site motifs were represented by sequences ^112^GRQGR↓L^117^ (*n* = 6), ^112^GKQGR↓L^117^ (*n* = 5), and ^112^ERQGR↓L^117^ (*n* = 1) that are typical for viruses of low virulence.

Pathogenicity evaluation was performed for eleven Ukrainian viruses using ICPI. The ICPI values ranged from 0.05 to 1.7 (Table 1). Four viruses had an ICPI value below 0.7, which characterizes them as lentogenic (avirulent) which is in agreement with the predictions based on deduced amino acid cleavage site sequences. ICPI values equal to or above 0.7 indicate a virulent strain. Virulent strains with ICPI values below 1.5 are mesogenic (moderate virulence) and those with values above 1.5 are velogenic NDV strains (3, 55, 57). Thus, six viruses were classified as mesogenic and one (pigeon/Ukraine/Kharkiv/23-01-/967/2013) as velogenic (3, 55).

### 3.3. Genotypic characterization

**Figure 2:**
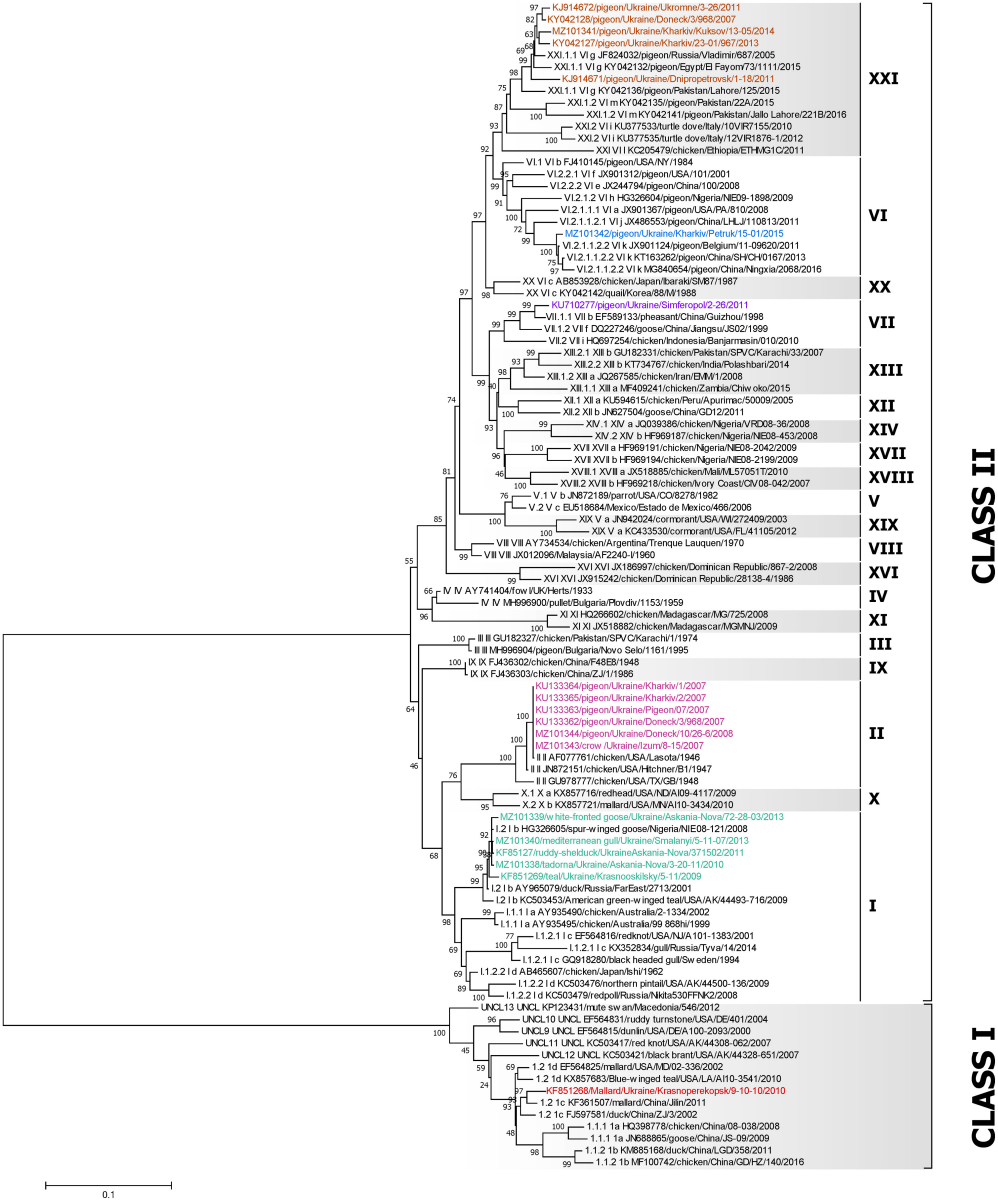
Phylogenetic analysis of NDV class I and II isolates based on the complete fusion gene sequences constructed with the maximum likelihood method, based on the general time-reversible model in MEGA v. 7.0.26. The percentage of trees in which the associated taxa clustered together in the bootstrap test (1,000 replicates) is shown next to the branches. The analysis involved 98 nucleotide sequences, 79 represent all class I and II sub-genotypes described by Dimitrov et al. (5) and 19 were collected from wild and synanthropic birds in Ukraine. All positions containing gaps and missing data were eliminated. There were a total of 1,661 positions in the final data set. The isolates used in this study are shown in colors. The Roman numerals presented in the taxa names in the phylogenetic tree represent the respective sub-genotype for each isolate, followed by the GenBank identification number, host name, country of isolation, strain designation, and year of isolation (if available).

A dataset of 79 complete F gene coding sequences of class I and II NDV isolates retrieved from GenBank was added to the 19 sequences from this study and used to construct a pilot tree for the preliminary identification of NDV genotypes as previously described (5). Based on the pilot tree, one NDV isolate belonged to class I and 18 to class II (Figure 2). Among class II isolates, five were of genotype I, six sequences were of genotype II, one was of genotype VI, one was of genotype VII, and five were of genotype XXI (Figure 2).

### 3.4. Genotype 1 isolates

**Figure 3:**
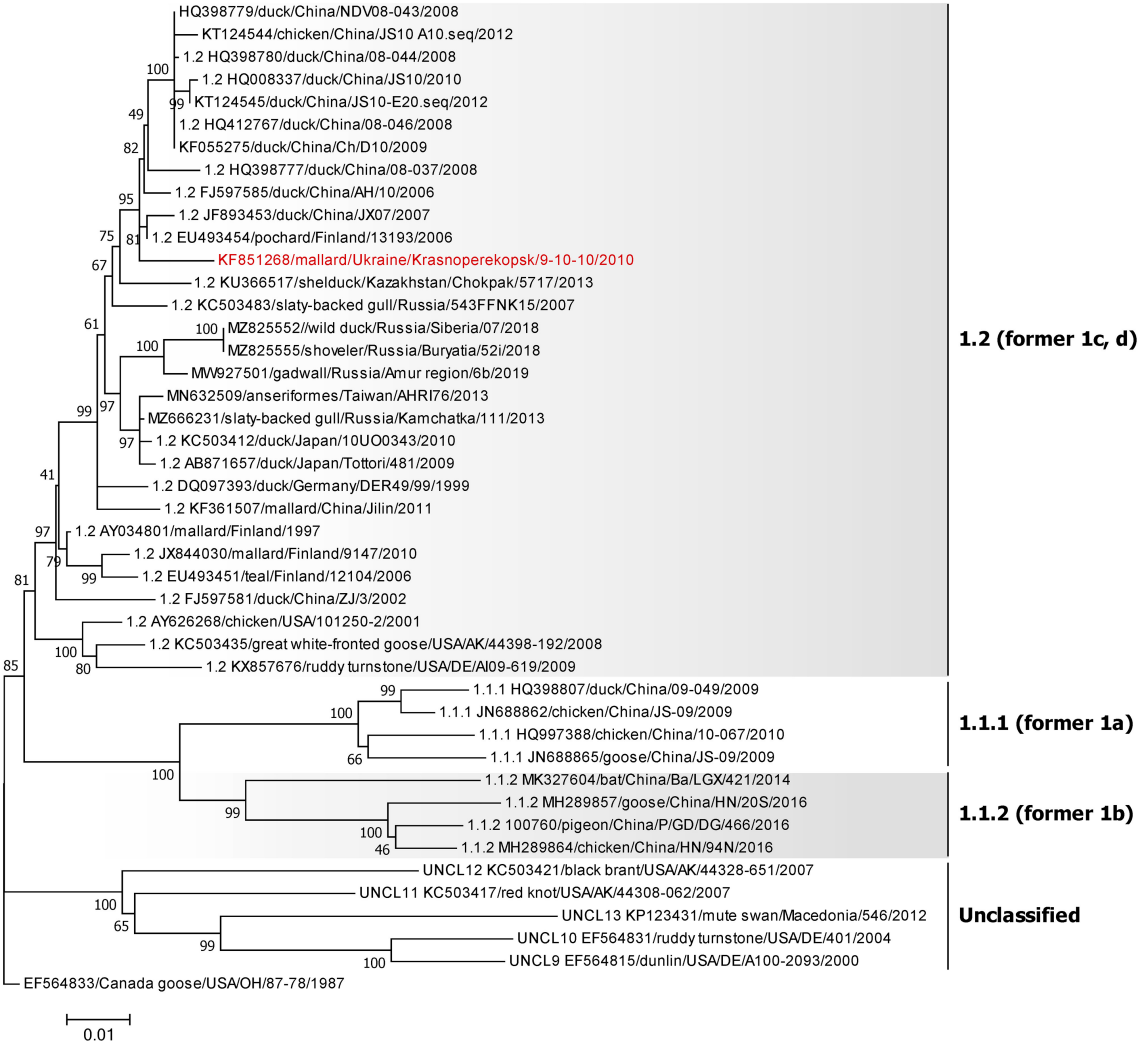
Phylogenetic analysis of NDV class I isolates based on the complete fusion gene sequences constructed with the maximum likelihood method with 1,000 bootstrap replicates. The analysis involved 44 nucleotide sequences. The tree was rooted to the oldest class I NDV isolate EF564833/Canada goose/USA/OH/78/1987. All positions containing gaps and missing data were eliminated. There were a total of 1,662 positions in the final data set. The isolates used in this study are shown in red. The Roman numerals on the right of the phylogenetic tree represent the respective sub-genotype for each isolate in accordance with the current classification (5) and the former nomenclature (58) is listed in parenthesis for reference.

The single NDV isolate of class I was collected from a mallard in AR Crimea in 2010 (KF851268) and belongs to sub-genotype 1.2 of class I (Figure 3), previously known as sub-genotype 1c (5, 58). The highest homology of this isolate is with the two strains—pochard in Finland from 2006 (EU493454) and duck in China from 2007 (JF893453), sharing 98.81% nucleotide identity.

### 3.5. Genotype I isolates

**Figure 4:**
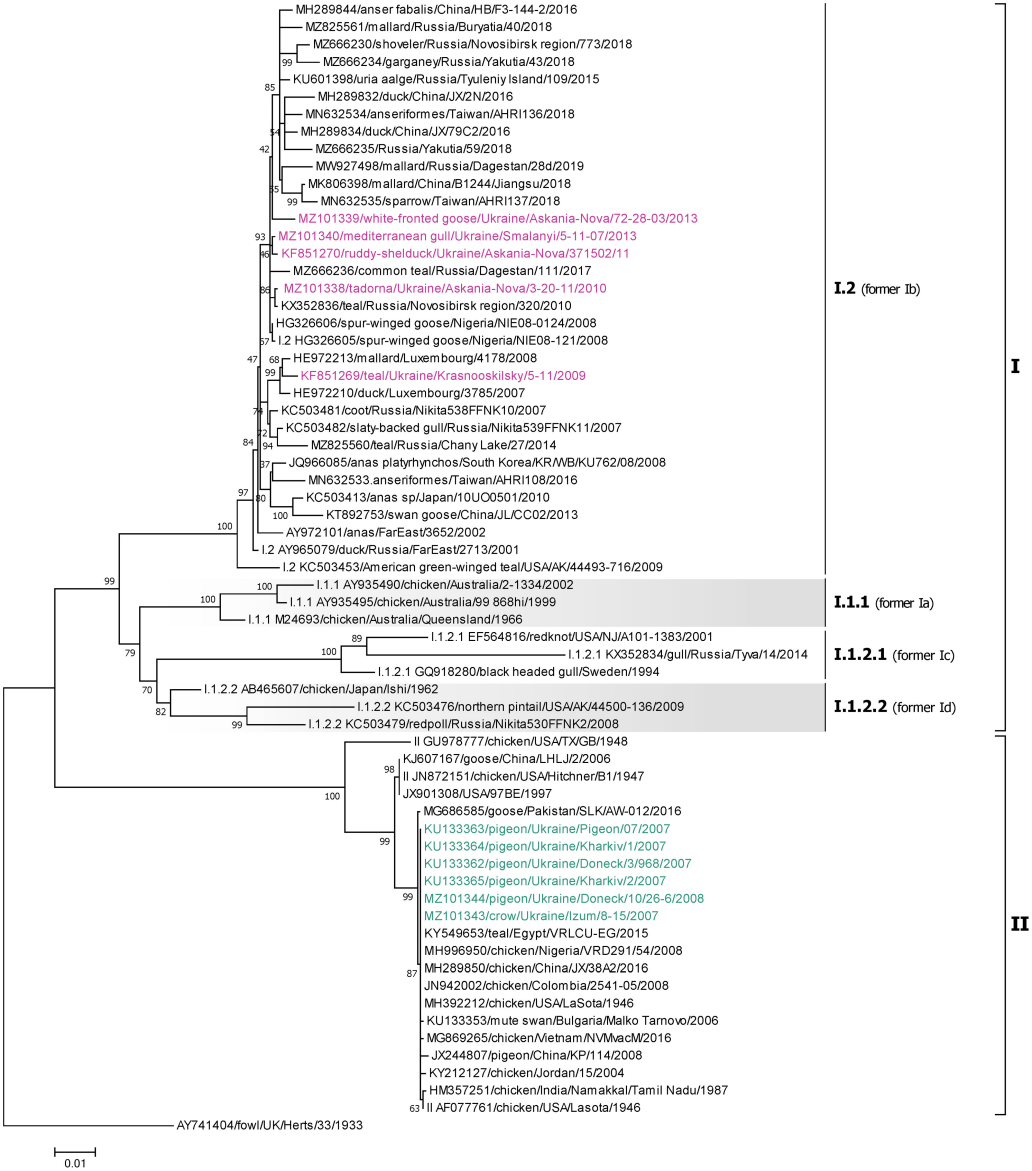
Phylogenetic analysis of NDV genotype I and II class II isolates based on the complete fusion gene sequences constructed with the maximum likelihood method with 1,000 bootstrap replicates. The analysis involved 65 nucleotide sequences (a sequence from genotype IV AY741404/fowl/UK/Herts/33/1933 was included as an outgroup). All positions containing gaps and missing data were eliminated. There were a total of 1,661 positions in the final data set. The isolates used in this study are shown in pink (genotype I) and teal (genotype II). The Roman numerals on the right of the phylogenetic tree represent the respective sub-genotype for each isolate in accordance with the current classification (5) and the former nomenclature (58) is listed in parenthesis for reference.

All five isolates of class II genotype I were classified as sub-genotype I.2 (Figure 4). These strains were collected from wild birds in the Kherson and Donetsk regions and shared between 98.50 and 99.82% nucleotide identity. Among them, the isolate collected from a white-fronted goose (MZ101339) had the highest nucleotide identity of 99.4% with the isolate collected from a Mediterranean gull (MZ101340) in 2013, which had an even higher nucleotide identity of 99.82% with another Ukrainian strain collected from ruddy shelduck (genus *Tadorna*) in 2011 (KF851270) (29). All these viruses were collected in the Kherson region. The fourth strain (MZ101338) from the Kherson region, isolated from ruddy shelduck in 2010, was most similar to an isolate originating from central Eurasia (Novosibirsk region) in 2010 (KX352836) (25) and shared 99.88% nucleotide identity. The only virus from the Donetsk region was collected from teal in 2009 (KF851269) and had the highest nucleotide homology with the strain isolated from mallard in Luxembourg in 2008 (HE972213) (59).

### 3.6. Genotype II isolates

All six NDV genotype II isolates (KU133362–KU133365, MZ101343, and MZ101344) were 100% identical (Figure 4). These isolates were collected from dead pigeons in Dnipro, Donetsk, and Kharkiv regions and a dead crow (genus *Corvus)* in the Kharkiv region in 2007 (23). These viruses were identical to the LaSota vaccine strain (MH392212) (60) and other vaccine strains isolated from different species of birds across different continents (37).

### 3.7. Genotype VI isolates

**Figure 5:**
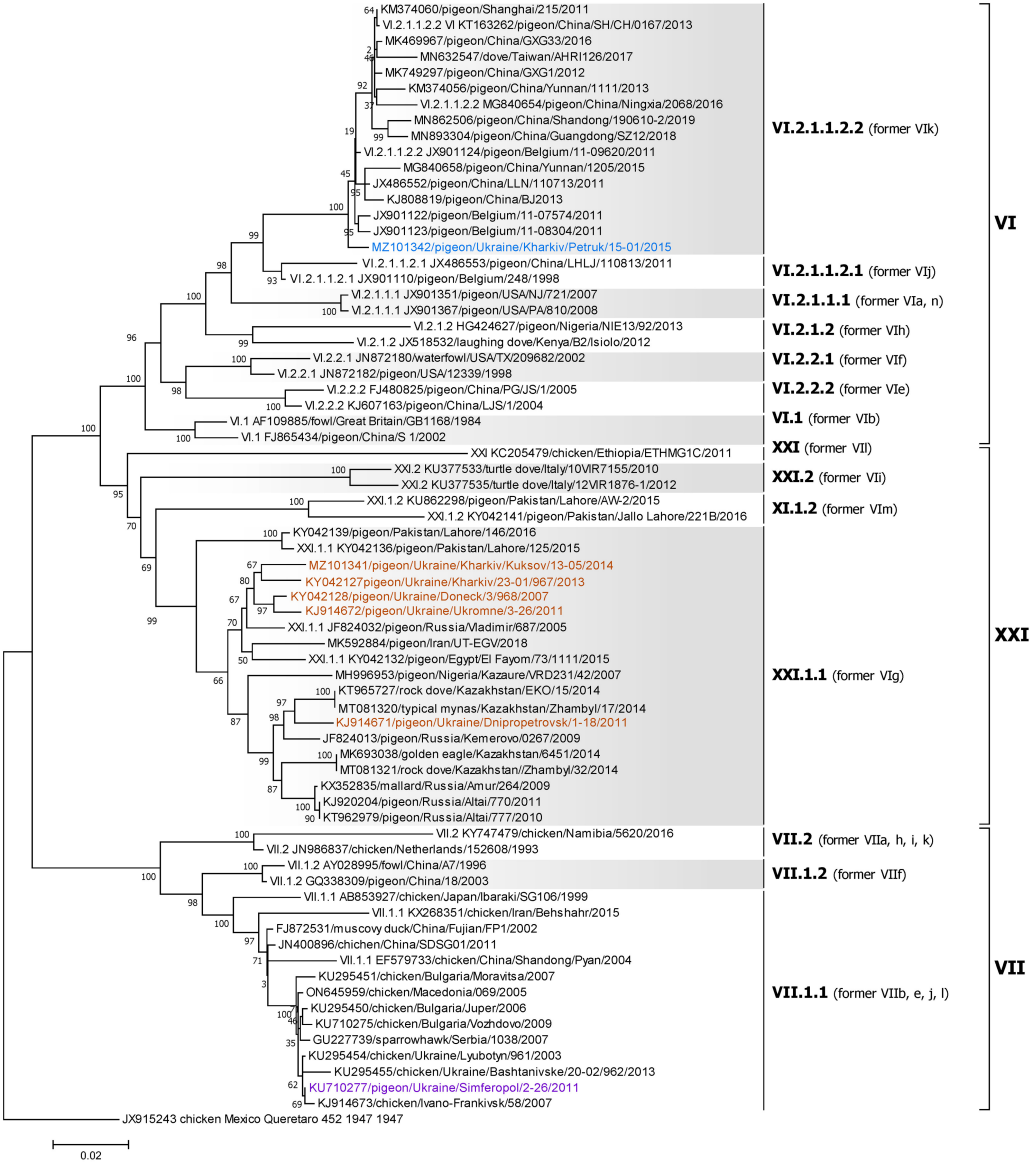
Phylogenetic analysis of NDV genotype VI, VII, and XXI class II isolates based on the complete fusion gene sequences constructed with the maximum likelihood method with 1,000 bootstrap replicates. The analysis involved 71 nucleotide sequences (a sequence from genotype XVI JX915243/chicken/Mexico/Queretaro/452/1947 was included as an outgroup). All positions containing gaps and missing data were eliminated. There were a total of 1,660 positions in the final data set. The isolates used in this study are shown in blue (genotype VI), purple (genotype VII), and brown (genotype XXI). The Roman numerals on the right of the phylogenetic tree represent the respective sub-genotype for each isolate in accordance with the current classification (5) and the former nomenclature (58) is listed in parenthesis for reference.

The single NDV genotype VI isolate was derived from a pigeon located in the Kharkiv region of Ukraine (MZ101342) and belongs to sub-genotype VI.2.1.1.2.2, formerly classified as sub-genotype VIk (Figure 5) (5, 58). The highest homology of this isolate is to a strain isolated from a pigeon in Belgium in 2011 (JX901124) (61), sharing 99.16% nucleotide identity.

### 3.8. Genotype VII isolates

The single NDV genotype VII isolate was derived from a pigeon located in Simferopol, AR Crimea (KU710277) and belongs to sub-genotype VII.1.1, formerly classified as sub-genotype VIId (Figure 5) (5, 58). The highest homology of this isolate is to a strain isolated from a chicken in the Kharkiv region of Ukraine in 2003 (KU295454) (44), sharing 99.88% nucleotide identity.

### 3.9. Genotype XXI isolates

All five genotype XXI isolates were detected in Ukrainian pigeons and belong to sub-genotype XXI.1.1, previously known as VIg (Figure 5) (5, 58). These strains were collected in Donetsk, Dnipro, Kharkiv, and AR Crimea regions and shared between 95.91 and 98.98% nucleotide identity. Of these, three (KJ914672, KY042127, and MZ101341), that were collected in Kharkiv and AR Crimea, had the highest homology to another Ukrainian strain (KY042128) isolated from a pigeon in the Donetsk region in 2007 (27), sharing 98.98, 98.13, and 98.07% nucleotide identity, respectively (27, 28). Another Ukrainian sub-genotype XXI.1.1 sequence from the Dnipro region (KJ914671) shared 98.01% homologies with two strains isolated from pigeons in Kazakhstan in 2014 (KT965727 and MT081320) (62).

## 4. Discussion

Our previous studies, as well as this one, provide essential information on the epidemiology of the NDV isolates from synanthropic and wild birds in Ukraine. In this study, we used Sanger sequencing to obtain complete F gene sequences of NDV in Ukraine from 2006 to 2015. Our study reports repeated NDV detection of sub-genotype I.2 in wild birds collected at the stopping points of migratory birds in Ukraine, as well as the first occurrence of sub-genotype VI.2.1.1.2 and the continuous presence of sub-genotypes II and XXI.1.1 in Ukrainian synanthropic birds. Additionally, we present the first publicly available complete NDV F gene from a crow (genus *Corvus*).

The Azov-Black Sea region of Ukraine is part of three transcontinental wild bird migration routes: the West Asia-East Africa, East Atlantic, and Black Sea-Mediterranean flyways (30, 31, 45). This region is comprised of areas for transit, stops during migration, and nesting for many bird species, which makes it one of the highly important regions in Eurasia for monitoring and studying the global circulation of NDV and predicting the emergence of new strains possibly transmitted by wild birds.

The single class I NDV isolate was detected from wild waterfowl at a major stopping point location in AR Crimea and belonged to the 1.2 sub-genotype, formerly known as 1c. This virus clustered together with strains also isolated from wild waterfowl of the order *Anseriformes* (family *Anatidae*) in China and Finland. The high identity between these isolates from Europe and Asia supports our hypothesis of intercontinental viral transmission by migratory birds.

All detected class II NDV isolates from wild waterfowl and shorebirds collected at the major stopping point locations of migratory routes in Ukraine, belonged to genotype I, which is consistent with our previous study (29, 63) and confirms the continuous threat of virus introduction from wild birds. All viruses were obtained from members of the orders *Anseriformes* (family *Anatidae*) and Charadriiformes (family *Laridae*). Of the five genotype I strains analyzed, all were classified as sub-genotype I.2. These isolates grouped together with isolates previously detected in different hosts at various locations, including (25, 29). Luxembourg, the North Caucasian, Siberian, and Far East Federal Districts of Russia, China, Taiwan, and Nigeria which confirms the intercontinental spread of the virus by migratory birds. Similar findings were reported in our previous studies where epidemiological connections of Avian Paramyxoviruses between Europe and Africa were shown (29). The high identity between isolates from wild birds in Europe and Asia, and their close phylogenetic relationship with strains from Africa, support our hypothesis of virus exchange along the Black Sea-Mediterranean and Asian-East African migratory flyways and highlight the possibility of intercontinental viral transmission.

Special attention should be given to the data obtained from the phylogenetic analysis of NDVs isolated from synanthropic birds in Ukraine. Six of these isolates, which were isolated from pigeons and a crow in three Eastern and Southern regions of Ukraine in 2007 and 2008, were classified as genotype II. To the best of our knowledge, there was no complete NDV F gene of any sub-genotype from crows in public databases prior to this study. However, partial F gene sequences of viruses of genotypes II isolated from crows in India in 2002 (AY339400) and Pakistan in 2017 (MN728799–MN728801) were available in the GenBank database (64). Interestingly, Ukrainian viruses were identical to the vaccine strain LaSota, which is widely used in Ukraine and around the world as a live vaccine against NDV (37). Industrial and backyard poultry farming is very developed in these Eastern regions of Ukraine. Furthermore, one of the last NDV outbreaks in Ukraine was previously recorded in one of those regions (Kharkiv region) in 2006 (3, 29). Because of that outbreak, a number of anti-epizootic measures were implemented, involving vaccination against NDV in industrial and backyard farms (including those where poultry had direct contact with synanthropic birds). We supposed the presence of a vaccine virus found in the synanthropic birds could be a result of contact with vaccinated poultry. In recent decades, the number of genotypes has increased. It is likely that the spread of a vaccine strain could contribute to an increase in the genetic diversity of NDV (32). Therefore, special attention must be paid to the distribution of vaccine strains in wild birds in order to understand the consequences of global vaccination.

NDV isolates of XXI genotype were identified in five dead pigeons with clinical signs of disease, which not only confirmed that sub-genotype XXI.1.1 is seemingly maintained in pigeons in the East of Ukraine (Donetsk and Kharkiv regions) from 2007 to 2014 (27) but also was detected for the first time in the South of Ukraine (Dnipro and Simferopol regions). Even though the isolates collected in Donetsk, Kharkiv, and AR Crimea regions grouped together, the isolate collected in the Dnipro region in 2011 was highly similar to isolates from pigeons and wild birds from genus *Acridotheres* (KT965727 and MT081320) from Kazakhstan in 2014 (62). This further highlights the possibility of continuous intercontinental viral spread. This may also indicate an additional link in the distribution of genotype XXI strains or its association with a host preference for pigeons. Interestingly, two different NDV sub-genotypes (II and XXI.1.1) were detected in a pigeon from Donetsk collected in 2007. This was possible due to the utilization of two different sets of primers to amplify the complete F gene of different NDV genotypes. However, this is rare, because in some cases we were not able to amplify a complete F gene even though APMV-1 was confirmed in these isolates by serological methods. We are speculating that it was due to acquired mutations in the NDV F gene which resulted in the primer's mismatch. Also, we can't exclude the possibility of the cross-reactivity of different serotypes by serology, which we previously observed and reported in an isolate collected from a white-fronted goose in the Kherson region in 2011 (51, 52). This isolate weakly cross-reacted with APMV-1 and APMV-7 antisera in serology, but we were unable to amplify NDV's F gene by utilizing the set of primers for routine NDV detection. However, random whole-genome next-generation sequencing allowed us to assemble the complete genome and discover a new serotype of APMV (named APMV-13), which explained the previous inability to amplify the complete F gene using primers specific for NDV. This highlights the need for implementing random next-generation sequencing as a routine diagnostic tool in order to better perceive the complete epidemiological situation.

To the best of our knowledge, only viruses of sub-genotypes II, VII.1.1, and XXI.1.1 have been previously reported to circulate in pigeons in Ukraine (23, 27, 44, 65). In this study, we report the first identification of genotype VI NDV in Ukraine. This virulent NDV isolate, classified as sub-genotype VI.2.1.1.2, was obtained from a deceased pigeon in the Kharkiv region in 2015.

To obtain a complete picture of the distribution of NDV along the migratory flyways and to determine all circulating genotypes among wild waterfowl, it is necessary to continue the annual monitoring of NDV in the Azov-Black Sea region, as one of the major stopping points for migratory birds. Additional data will help to assess the degree of involvement of wild birds in the spread of virulent strains, that can be especially dangerous for poultry production. Further monitoring of NDV in synanthropic bird species will provide useful data for the study of vaccine strains widespread globally.

## Data availability statement

The datasets presented in this study can be found in online repositories. The names of the repository/repositories and accession number(s) can be found below: https://www.ncbi.nlm.nih.gov/genbank/, MZ101338, MZ101339, MZ101340, MZ101341, MZ101342, MZ101343, MZ101344, KF851268, KF851270, KJ914671, KJ914672, KU710277, KF851269, KU133362, KU133363, KU133364, KU133365, KY042127, and KY042128.

## Ethics statement

The animal study was reviewed and approved by the Institutional Animal Care and Use Committee of the National Scientific Center Institute of Experimental and Clinical Veterinary Medicine.

## Author contributions

Conceptualization: IG, AG, DM, and CA. Funding acquisition: CA and BS. Project administration and coordination: CA, AG, and DM. Methodology: IG. Sample preparation: IG, VB, OS, OR, NM, OM, and OK. Data curation and formal analyses: IG, KD, PM, and CA. Writing—original draft: IG and DM. Writing—review and editing: PM, CA, and MP-J. All authors have read and agreed to the published version of the manuscript.

## Supplementary material

Supplementary Table S1 Number of samples of biological material collected from wild birds of different species in different regions of Ukraine from 2006 to 2015.

Supplementary Table S2 Number of samples of biological material collected from synanthropic birds of different species in different regions of Ukraine from 2006 to 2015.
