# Supplementary material for: Genetic diversity of Newcastle disease viruses circulating in wild and synanthropic birds in Ukraine between 2006 and 2015
Source: Front Vet Sci. 2023 Jan 19;10:1026296. doi: 10.3389/fvets.2023.1026296 (PMC9893288; doi:10.3389/fvets.2023.1026296)
Supplement: Supplementary Table S1 — Number of samples of biological material collected from wild birds of different species in different regions of Ukraine from 2006 to 2015. [file Table_1.DOCX]

**Table S1.** Number of samples of biological material collected from wild birds of different species in different regions of Ukraine from 2006 to 2015

| **Birds Species** | | **Regions of Ukraine** | | | | | | | | | | | |
| --- | --- | --- | --- | --- | --- | --- | --- | --- | --- | --- | --- | --- | --- |
|  |  | **AR Crimea** | **Zaporizhzhya** | **Mykolaiv** | **Odesa** | | **Kherson** | **Kharkiv** | **Donetsk** | | **Total amount** | | |
| **Anseriformes** | | | | | | | | | | | | | |
| Shelduck *Tadorna tadorna* | | 152 | 324 |  | | 78 | 415 |  | |  | | 969 |  |
| White-fronted Goose  *Anser albifrons* | | 430 |  |  | | 565 | 4,687 | 120 | |  | | 5,802 |  |
| Greylag Goose  *Anser anser* | |  | 75 |  | | 334 | 38 |  | |  | | 447 |  |
| Branta ruficollis *Rufibrenta ruficollis* | | 130 |  |  | |  | 385 |  | |  | | 515 |  |
| Mallard *Anas platyrhynchos* | | 500 | 350 | 54 | | 55 | 1,800 | 30 | | 5 | | 2,794 |  |
| Bewick`s Swan *Cygnus bewickii* | | 10 |  |  | | 480 |  |  | |  | | 490 |  |
| Whooper Swan *Cygnus cygnus* | | 56 |  | 15 | | 479 | 38 |  | |  | | 588 |  |
| Mute Swan *Cygnus olor* | | 40 |  |  | | 61 | 79 |  | |  | | 180 |  |
| Gadwal *Anas strepera* | | 3 |  |  | |  |  |  | |  | | 3 |  |
| Ruddy Shelduck *Tadorna ferruginea* | |  | 70 |  | |  | 2,350 |  | |  | | 2,420 |  |
| Pochard *Aythya ferina* | | 2 |  |  | | 15 |  |  | |  | | 17 |  |
| Wigeon *Anas penelope* | | 19 |  |  | | 97 | 150 |  | |  | | 266 |  |
| Red-crested Pochard *Netta rufina* | | 3 | 5 |  | |  |  |  | |  | | 8 |  |
| Garganey *Anas querquedula* | | 4 | 67 |  | | 7 | 48 |  | |  | | 135 |  |
| Teal *Anas crecca* | | 119 | 76 |  | | 48 | 76 |  | | 9 | | 319 |  |
| Pintail *Anas acuta* | | 3 |  |  | |  |  |  | |  | | 3 |  |
| Shoveler *Anas clypeata* | | 4 |  |  | |  | 50 |  | | 2 | | 56 |  |
| Red-breasted Merganser *Mergus serrator* | |  |  | 1 | |  |  |  | |  | | 1 |  |
| **Charadriiformes** | | | | | | | | | | | | | |
| Snipe *Gallinago gallinago* | | 1 | 2 |  | |  |  |  | |  | | 3 |  |
| Jack Snipe *Lymnocryptes minimus* | | 1 | 1 |  | |  |  |  | |  | | 2 |  |
| Ruff *Phylomachus pugnax* | | 29 | 273 |  | | 12 | 55 |  | |  | | 369 |  |
| Wood Sandpiper *Tringa glareola* | |  | 45 |  | |  |  |  | |  | | 45 |  |
| Greenshank *Tringa nebularia* | | 16 | 6 |  | |  |  |  | |  | | 22 |  |
| Redshank *Tringa totanus* | | 14 | 4 |  | |  |  |  | |  | | 18 |  |
| Green Sandpiper *Tringa ochropus* | |  | 5 |  | |  |  |  | |  | | 5 |  |
| Marsh Sandpiper *Tringa stagnatilis* | |  | 1 |  | |  |  |  | |  | | 1 |  |
| Spotted Redshank *Tringa erythropus* | | 7 |  |  | |  |  |  | |  | | 7 |  |
| White-winged Black Tern *Chlidonias leucopterus* | |  | 44 |  | |  |  |  | |  | | 44 |  |
| Caspian Tern *Hydroprogne caspia* | | 65 |  |  | |  |  |  | |  | | 65 |  |
| Little Tern *Sterna albifrons* | |  | 5 |  | |  |  |  | |  | | 5 |  |
| Common Tern *Sterna hirundo* | |  | 55 |  | | 21 | 1 |  | |  | | 77 |  |
| Sandwich Tern *Thalasseus sandvicensis* | |  | 5 |  | |  | 19 |  | |  | | 24 |  |
| Gull-billed Tern *Gelochelidon nilotica* | | 10 | 10 |  | |  | 75 |  | |  | | 95 |  |
| Black-winged Stilt *Himantopus himantopus* | |  | 4 |  | |  |  |  | |  | | 4 |  |
| Yellow-legged Gull *Larus cachinnans* | | 45 | 309 | 90 | | 488 | 376 |  | |  | | 1,308 |  |
| Black-headed Gull *Larus ridibundus* | | 60 | 297 |  | | 155 | 103 |  | |  | | 615 |  |
| Great Black-headed Gull *Larus ichtyaetus* | |  |  |  | |  | 2 |  | |  | | 2 |  |
| Little Gull *Larus minutus* | |  | 121 |  | |  |  |  | |  | | 121 |  |
| Mediterranean Gull *Larus melanocephalus* | | 2 |  |  | |  | 436 |  | |  | | 438 |  |
| Common Gull *Larus canus* | | 19 | 7 |  | |  | 55 |  | |  | | 81 |  |
| Slender-billed Gull *Larus genei* | | 5 | 296 |  | |  | 16 |  | |  | | 317 |  |
| Oystercatcher *Haematopus ostralegus* | | 1 | 4 |  | |  |  |  | |  | | 5 |  |
| Ringed Plover *Charadrius hiaticula* | |  | 2 |  | |  |  |  | |  | | 2 |  |
| Kentish Plover *Charadrius alexandrinus* | |  | 5 |  | |  | 1 |  | |  | | 6 |  |
| Sanderling *Calidris alba* | |  |  |  | |  | 3 |  | |  | | 3 |  |
| Temminck`s Stint *Calidris temminckii* | |  | 3 |  | |  |  |  | |  | | 3 |  |
| Broad-billed Sandpiper *Limicola falcinellus* | | 13 | 4 |  | |  |  |  | |  | | 17 |  |
| Little Stint *Calidris minuta* | | 39 | 12 |  | |  |  |  | |  | | 48 |  |
| Curlew Sandpiper *Calidris ferruginea* | | 271 | 102 |  | |  |  |  | |  | | 372 |  |
| Dunlin *Calidris alpina* | | 271 | 84 |  | | 27 | 111 |  | |  | | 493 |  |
| Grey Plover *Pluvialis squatarola* | | 17 | 27 |  | |  | 4 |  | |  | | 48 |  |
| Avocet *Recurvirostra avosetta* | | 20 | 31 |  | |  |  |  | |  | | 51 |  |
| Collared Pratincole *Glareola pratincola* | |  | 1 |  | |  |  |  | |  | | 1 |  |
| Black-winged Pratincole *Glareola nordmanni* | |  | 5 |  | |  |  |  | |  | | 5 |  |
| Common Sandpiper *Actitis hypoleucos* | |  | 5 |  | |  |  |  | |  | | 5 |  |
| Lapwing *Vanellus vanellus* | |  | 2 |  | |  |  |  | |  | | 2 |  |
| Curlew *Numenius arquata* | | 3 |  |  | |  |  |  | |  | | 3 |  |
| Bar-tailed Godwit *Limosa lapponica* | | 1 |  |  | |  |  |  | |  | | 1 |  |
| **Ciconiiformes** | | | | | | | | | | | | | |
| Purple Heron *Ardea purpurea* | |  | 1 |  | |  |  |  | |  | | 1 |  |
| Grey Heron *Ardea cinerea* | | 4 | 35 |  | | 10 |  |  | |  | | 49 |  |
| Great Whitе Egret *Egretta alba* | |  | 12 |  | | 21 | 1 |  | |  | | 34 |  |
| Little Egret *Egretta garzetta* | |  |  |  | | 1 |  |  | |  | | 1 |  |
| White Stork *Ciconia ciconia* | |  |  |  | | 1 |  |  | |  | | 1 |  |
| Night Heron *Nycticorax nycticorax* | |  |  |  | | 40 |  |  | |  | | 40 |  |
| Glossy Ibis *Plegadis falcinellus* | |  |  |  | | 9 | 1 |  | |  | | 10 |  |
| **Columbiformes** | | | | | | | | | | | | | |
| Woodpigeon *Columba palumbus* | |  |  |  | | 239 |  |  | |  | | 239 |  |
| **Coraciiformes** | | | | | | | | | | | | | |
| Kingfisher *Alcedo atthis* | |  | 3 |  | |  |  |  | |  | | 3 |  |
| **Falconiiformes** | | | | | | | | | | | | | |
| Lesser Kestrel *Falco naumanni* | |  | 1 |  | |  |  |  | |  | | 1 |  |
| Buzzard *Buteo buteo* | |  |  |  | | 1 |  |  | |  | | 1 |  |
| **Galliformes** | | | | | | | | | | | | | |
| Partridge *Perdix perdix* | | 4 |  |  | |  |  |  | |  | | 4 |  |
| **Gruiformes** | | | | | | | | | | | | | |
| Great Bustard *Otis tarda* | |  |  |  | |  | 35 |  | |  | | 35 |  |
| Crane *Grus grus* | | 75 | 65 |  | |  | 276 |  | |  | | 416 |  |
| Moorhen *Gallinula chloropus* | |  | 2 |  | |  |  |  | |  | | 2 |  |
| Water Rail *Rallus aquaticus* | |  | 1 |  | |  |  |  | |  | | 1 |  |
| Little Crake *Porzana parva* | |  | 2 |  | |  |  |  | |  | | 2 |  |
| Coot *Fulica atra* | | 28 | 47 | 50 | | 16 | 76 |  | | 1 | | 218 |  |
|  | **Passeriformes** | | | | | | | | | | | | |
| Jackdaw *Corvus monedula* | |  |  |  | |  | 25 |  | |  | | 25 |  |
| Tree Sparrow *Passer montanus* | |  |  |  | |  | 5 |  | |  | | 5 |  |
| House Sparrow *Passer domesticus* | |  |  |  | | 52 |  |  | |  | | 52 |  |
| Rook *Corvus frugilegus* | | 1 | 23 |  | | 355 | 60 |  | |  | | 439 |  |
| Calandra Lark *Melanocorypha calandra* | |  |  |  | |  | 60 |  | |  | | 60 |  |
| Chaffinch *Fringilla coelebs* | |  |  |  | |  | 2 |  | |  | | 2 |  |
| Reed Bunting *Emberiza schoeniclus* | | 15 | 1 |  | | 1 | 5 |  | |  | | 22 |  |
| Sand Martin *Riparia riparia* | |  | 13 |  | |  |  |  | |  | | 13 |  |
| Swallow *Hirundo rustica* | |  | 8 |  | | 2 |  |  | |  | | 10 |  |
| Great Reed Warble *Acrocephalus arundinaceus* | |  | 11 |  | |  |  |  | |  | | 11 |  |
| Sedge Warbler *Acrocephalus schoenobaenus* | |  | 1 |  | |  |  |  | |  | | 1 |  |
| Reed Warbler *Acrocephalus scirpaceus* | |  | 4 |  | |  |  |  | |  | | 4 |  |
| Pied Wagtai *Motacilla alba* | |  | 1 |  | | 9 |  |  | |  | | 10 |  |
| Yellow Wagtail *Motacilla flava* | |  | 1 |  | |  |  |  | |  | | 1 |  |
| Corn Bunting *Emberiza calandra* | |  |  |  | | 4 |  |  | |  | | 4 |  |
| Starling *Sturnus vulgaris* | | 126 | 27 |  | |  |  |  | |  | | 153 |  |
| Goldfinch *Carduelis carduelis* | |  |  |  | | 23 |  |  | |  | | 23 |  |
| Bearded Tit *Panurus biarmicus* | |  | 8 |  | |  |  |  | |  | | 8 |  |
| Long-tailed Tit *Aegithalos caudatus* | |  |  |  | | 1 |  |  | |  | | 1 |  |
| Magpie *Pica pica* | | 35 |  |  | | 2 |  |  | |  | | 37 |  |
| Icterine Warbler *Hippolais icterina* | |  | 1 |  | |  |  |  | |  | | 1 |  |
| Savi`s Warbler *Locustella luscinioides* | |  | 1 |  | |  |  |  | |  | | 1 |  |
| Lesser Whitethroat *Sylvia curruca* | |  |  |  | | 3 |  |  | |  | | 3 |  |
| Song Thrush *Turdus philomelos* | | 1 |  |  | |  |  |  | |  | | 1 |  |
| Blackbird *Turdus merula* | | 1 |  |  | |  |  |  | |  | | 1 |  |
| **Pelecaniformes** | | | | | | | | | | | | | |
| Cormorant *Phalacrocorax carbo* | |  |  |  | | 54 | 103 |  | |  | | 157 |  |
| **Strigiformes** | | | | | | | | | | | | | |
| Long-eared Owl *Asio otus* | |  | 1 |  | |  |  |  | |  | | 1 |  |
| **Total** | | **2,675** | **3,014** | **210** | | **3,766** | **12,022** | **150** | | **17** | | **21,854** |  |
