# Supplementary material for: Genetic diversity of Newcastle disease viruses circulating in wild and synanthropic birds in Ukraine between 2006 and 2015
Source: Front Vet Sci. 2023 Jan 19;10:1026296. doi: 10.3389/fvets.2023.1026296 (PMC9893288; doi:10.3389/fvets.2023.1026296)
Supplement: Supplementary Table S2 — Number of samples of biological material collected from synanthropic birds of different species in different regions of Ukraine from 2006 to 2015. [file Table_2.DOCX]

**Table S2.** Number of samples of biological material collected from synanthropic birds of different species in different regions of Ukraine from 2006 to 2015

| **Species** | **Region** | **Number of samples** |
| --- | --- | --- |
| **2007** | | |
| Pigeon *Columba livia* | Kharkiv, Ukraine | 2 |
|  | Dnipro, Ukraine | 1 |
|  | Donetsk, Ukraine | 1 |
| Rook *Corvus frugilegus* | Kharkiv, Ukraine | 1 |
| **2008** | | |
| Pigeon *Columba livia* | Donetsk, Ukraine | 10 |
|  | Kharkiv, Ukraine | 2 |
| **2009** | | |
| Pigeon *Columba livia* | Donetsk, Ukraine | 6 |
|  | Kharkiv, Ukraine | 1 |
| **2010** | | |
| Pigeon *Columba livia* | Donetsk, Ukraine | 2 |
|  | Kharkiv, Ukraine | 1 |
| **2011** | | |
| Pigeon *Columba livia* | Donetsk, Ukraine | 1 |
|  | Kharkiv, Ukraine | 4 |
|  | Dnipro, Ukraine | 1 |
|  | AR Crimea, Ukraine | 2 |
| **2012** | | |
| Pigeon *Columba livia* | Kharkiv, Ukraine | 4 |
| **2013** | | |
| Pigeon *Columba livia* | Kharkiv, Ukraine | 7 |
|  | Odesa, Ukraine | 4 |
|  | AR Crimea, Ukraine | 1 |
| **2014** | | |
| Pigeon *Columba livia* | Kharkiv, Ukraine | 8 |
|  | Odesa, Ukraine | 1 |
| **2015** | | |
| Pigeon *Columba livia* | Kharkiv, Ukraine | 3 |
|  | Dnipro, Ukraine | 2 |
|  | Donetsk, Ukraine | 3 |
|  | Odesa, Ukraine | 2 |
| **Total** |  | **70** |
